# A Parallel-Arm Randomized Controlled Trial to Assess the Effects of a Far-Infrared-Emitting Collar on Neck Disorder 

**DOI:** 10.3390/ma8095279

**Published:** 2015-09-01

**Authors:** Yung-Sheng Lin, Kuo-Sheng Hung, Ben-Yi Liau, Chih-Hui Yang, Aiga Yang, Keng-Shiang Huang

**Affiliations:** 1 Department of Chemical Engineering, National United University, Miaoli 36063, Taiwan; E-Mail: aigayang@gmail.com; 2 Department of Neurosurgery, Taipei Medical University-Wan Fang Hospital, Taipei 11696, Taiwan; E-Mail: kshung25@gmail.com; 3 Graduate Institute of Injury Prevention and Control, Taipei Medical University, Taipei 11031, Taiwan; 4 Department of Biomedical Engineering, Hungkuang University, Taichung 43302, Taiwan; E-Mail: byliau@sunrise.hk.edu.tw; 5 Department of Biological Science and Technology, I-Shou University, Kaohsiung 82445, Taiwan; E-Mail: chyang@isu.edu.tw; 6 The School of Chinese Medicine for Post-Baccalaureate, I-Shou University, Kaohsiung 82445, Taiwan; E-Mail: huangks@isu.edu.tw

**Keywords:** far-infrared ray, collar, neck, pain

## Abstract

The purpose of this study is to assess the beneficial effects of a far-infrared-emitting collar (FIRC) on the management of neck disorders. A neck disorder is generalized as neck muscle pain and its relative mental disorders because the etiologies of the neck’s multidimensional syndrome are either muscle impairment or psychiatric distress. This is the first study to determine the efficacy of a FIRC by evaluating objective physical evidence and psychometric self-reports using a parallel-arm randomized sham-controlled and single-blinded design. In this trial, 60 participants with neck disorders were observed at baseline and post-intervention. Compared to the placebo group after a 30-min intervention, the FIRC demonstrated a statistically significant biological effect in elevating skin temperature and promoting blood circulation with *p*-values 0.003 and 0.020, respectively. In addition, FIRC application significantly reduced neck muscle tension, relieved pain, ameliorated fatigue, improved depression, and decreased anxiety. The FIRC could therefore be a potential treatment for neck disorders.

## 1. Introduction

Far-infrared (FIR) ray is an invisible light with an electromagnetic radiation wavelength of 3–1000 μm, as defined by the division scheme of the International Commission on Illumination (CIE). Over the past two decades, the biological benefits of FIR have been studied and have served as a treatment of vascular-related disorders [1,2,3]. At a wavelength ranging between 4 and 14 μm [4,5,6], the FIR can trigger both thermal and non-thermal effects at the molecular level to affect the health of a whole living organism [1,2,3,4,5,6,7,8,9,10,11,12,13,14]. Through the resonance absorption process of the human body, the FIR can operate from inside tissues to promote body metabolism more effectively than a single heating of the skin surface. The FIR has been proven effective in healing wounds [7], relieving pain [8,9,10,11], ameliorating fatigue [10,11,12,13], and improving mood [5,9,11,12,13,14].

FIR has generally been evaluated as an effective, safe, and non-pharmacologic alternative for promoting human health [8,9,10,11,12,13,14]. However, the multiple beneficial effects and the mechanism of FIR on pain relief and the modification of mental disorders remain poorly understood. Most studies undertook FIR therapy as a complementary treatment, only eliciting self-rating evaluations and lacking objective physical evidence. In particular, on account of trial limitations, FIR sauna treatments lacked a placebo to validate the tests. When managing pain relief assessment, the comprehensive observations of psychophysiological factors and physiological response realities comparing with the sham treatment control are needed. A high interest still exists in these aspects to clarify the efficacy of FIR therapy.

“A pain in the neck” is not only a proverbial saying. Musculoskeletal pain in the neck, with accompanying fatigue and mood syndromes, is a common disorder. Epidemiological studies show that approximately 26% of the general population suffers from a neck disorder every year [15,16,17]. From school children to adults, individuals may experience this unpleasant disorder [16,17,18,19]. People with work-related stress and muscular tension are particularly affected by neck disorders, resulting in damage to individual welfare and major socioeconomic impact [15,20]. Clinical treatment of neck disorders is difficult because the structure of the neck is highly complex, and pathogenesis may be unclear. Disorders may derive from a poor physical posture that leads to muscular fatigue or pain. Alternatively, neck pain may arise primarily from a nonorganic problem, such as work-related stress or depression. Therefore, identifying the local pathological causes of neck disorders and providing suitable treatment is not a simple task [17,21]. For those with neck disorders, concern may exist over the prolonged use of medicines, which may fail to provide a cure and also produce a range of side effects [22]. In such circumstances, some alternative strategies are proposed. A tailored personalized exercise program can have a high value (with respect to a general approach) to reduce and/or prevent neck pain [23,24]. Besides, drug-free FIR therapy appears to be a good potential treatment as a complementary or alternative medication for the management of neck disorders.

This is the first study to evaluate FIR therapy by a FIR-emitting collar (FIRC) for the neck disorders. The aim was to study the effects of FIRC compared to a sham collar on the physical and mental health of those suffering from neck disorders. To overcome the limitations of prior studies, we not only elicited psychometric self-assessment outcomes, but also collected objective evidence. In addition, sham collars were used as a placebo to validate any beneficial findings. 

## 2. Results and Discussion

Table 1 summarizes the results in this study. The information of participants for the whole groups, the control group, and the intervention group, and the statistical analysis are also included. 

### 2.1. Muscle Hardness

**Figure 1:**
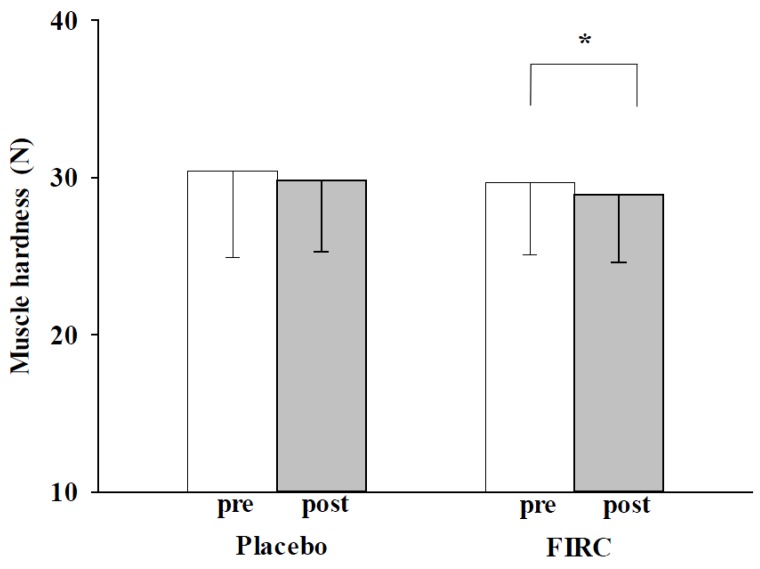
Changes in neck muscle hardness of placebo and FIRC groups before and after intervention. Asterisk indicates a significant difference.

Neck muscle hardness in the FIRC group exhibited a significant decrease from baseline to post-intervention (29.8 ± 4.5 N *vs*. 28.9 ± 4.3 N; *p* < 0.05), whereas no significant difference was found for the placebo group (Figure 1). In this study, the muscle hardness barometer was used by the same investigator to measure neck muscle tone validly. Having the same investigator use the tool on all patients would help to control potential errors between examiners. Previous studies have mentioned that a tender point of neck pain is located at the midpoint between the seventh cervical vertebra and the acromion. We therefore applied the muscle hardness barometer to measure the point at the trapezius muscles [25].

Neck muscle rigidity is considered to be related to musculoskeletal pain and mental tenseness, whether a physical syndrome leads to neck pain accompanied by mood distress, or muscular tension causing neck pain arises from psychological factors [26,27,28,29,30,31]. Neck disorders have commonly been found to be related to poor posture; working at a computer, playing video games, texting, reading, engaging in assembly-line operation, and so on. Having a poor neck posture for an extended period would cause neck muscle fatigue, stiffness, impairment, and result in pain [26,27,29,30]. Likewise, stress, depression, anxiety, and other mood disorders have been found to have comorbidity with neck muscle tension. In direct opposition to this, the degree of hardness of neck muscles can be a predisposing factor for pain-related mood disorders [22,31]. Above all, postural neck pain and mood disorders are associated with muscle status. In this study, we found that after wearing the FIRC for 30 min, the participants’ neck muscle tension had decreased significantly. In contrast, the placebo group wearing sham collars showed no significant decrease. This result implies that the FIRC may improve neck disorders by alleviating neck muscle hardness.

**Table 1:** Comparison of measured results in the control group and the intervention group.

Group		Placebo Group				FIR group				
Measurement		Pre	Post	Difference (Δ*P*)	*P* Value (pre *vs.* post)	Pre	Post	Difference (Δ*F*)	*P* Value (pre *vs.* post)	*P* Value (Δ*P vs.* Δ*F*)
Muscle hardness (N)		30.43 ± 5.5	29.7 ± 4.6	−0.7 ± 3.7	0.190	29.8 ± 4.5	28.9 ± 4.3	−1.2 ± 2.8	0.026 *	0.412
Skin temperature (°C)		34.0 ± 0.6	35.1 ± 0.9	1.1 ± 0.9	0.001 *	34.0 ± 0.7	35.9 ± 0.6	1.8 ± 0.9	0.001 *	0.003 *
Blood flow (PU)		93.5 ± 23.8	174.4 ± 65.7	80.9 ± 63.0	0.001 *	96.2 ± 21.3	228.9 ± 95.8	132.6 ± 96.8	0.001 *	0.020 *
Visual analogue scale	Pain	3.6 ± 2.3	2.1 ± 1.8	−1.5 ± 1.3	0.001 *	3.8 ± 2.2	2.0 ± 1.9	−1.7 ± 1.5	0.001 *	0.433
	Anxiety	2.8 ± 2.3	1.7 ± 2.1	−1.1 ± 1.9	0.004 *	3.5 ± 2.3	2.0 ± 1.8	−1.8 ± 1.7	0.001 *	0.077
	Depression	2.2 ± 2.2	1.3 ± 1.8	−0.9 ± 1.4	0.002 *	3.2 ± 2.3	1.7 ± 1.7	−1.4 ± 1.6	0.001 *	0.122
	Fatigue	4.6 ± 2.5	2.5 ± 2.1	−2.1 ± 2.2	0.001 *	5.2 ± 2.6	2.9 ± 2.2	−2.4 ± 2.1	0.001 *	0.615

Placebo group: 12 males & 18 females, age: 19.7 ± 1.7 years; FIRC group: 13 males & 17 females, age: 19.6 ±2.3 years. A value of *p* < 0.05 was considered significant difference and labelled *.

### 2.2. Temperature 

**Figure 2:**
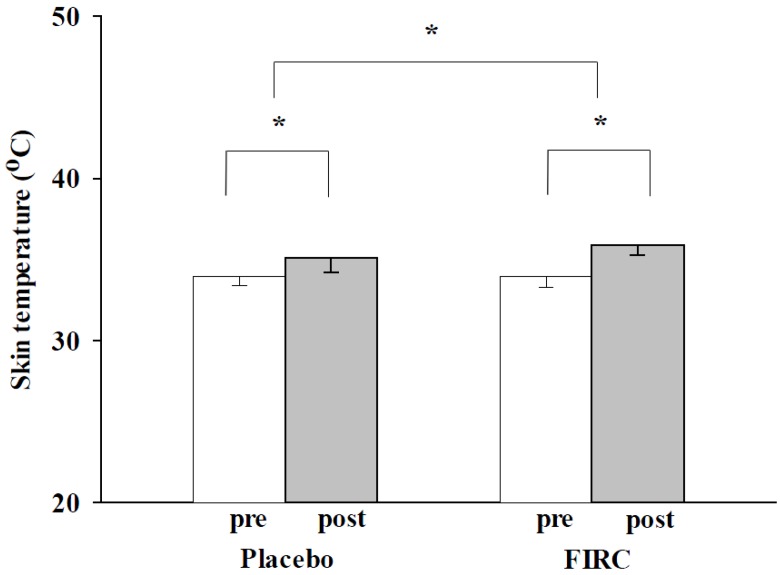
Changes in neck skin temperature of placebo and FIRC groups before and after intervention. Asterisk indicates a significant difference.

The collar intervention had a significant warming effect on both the FIRC group and placebo group (results shown in Figure 2; FIRC group: pre-34.0 ± 0.7 °C *vs*. post-35.9 ± 0.6 °C, *p* < 0.05; Placebo group: pre-34.0 ± 0.6 °C *vs*. post-35.1 ± 0.9 °C, *p* < 0.05). In a comparison of the degree of warming between the groups, the FIRC group neck temperature was elevated more than that of the placebo group (*p* < 0.05). Normal increase of skin temperature has been postulated to activate blood circulation and metabolism [2,32].

It is known that a hot pack or heat device with FIR can keep the body warm [8,9,10,13,14]. Hot devices provided thermal energy in this study, and both groups experienced this beneficial effect [8]. The prior study indicated local thermal stimulation generated heat energy to a depth of approximately 10 mm in the underlying subcutaneous tissue [32]. Our evidence further demonstrated that the FIRC could keep the neck warmer than a single heated collar (the placebo collar). This may be because of the unique resonance absorption process of FIR radiation, which can reach deeper into the skin of the neck than single thermal energy. In the meantime, FIR energy was preserved deeply under the skin. Neck temperature is an indicator of neck circulatory hemodynamics [33]; the application of FIRC significantly elevated neck temperature, and was further expected to increase neck circulation. This implied that FIRC application may alleviate neck disorders by promoting the metabolism of the neck region better than the sham collar. In addition, the temperature increase from the FIRC is safe because none of the participants reported any discomfort, burns, or side effects. The warming capability of the FIRC was combined with the alleviation of neck muscle hardness to improve the neck disorders.

### 2.3. Blood Flow

**Figure 3:**
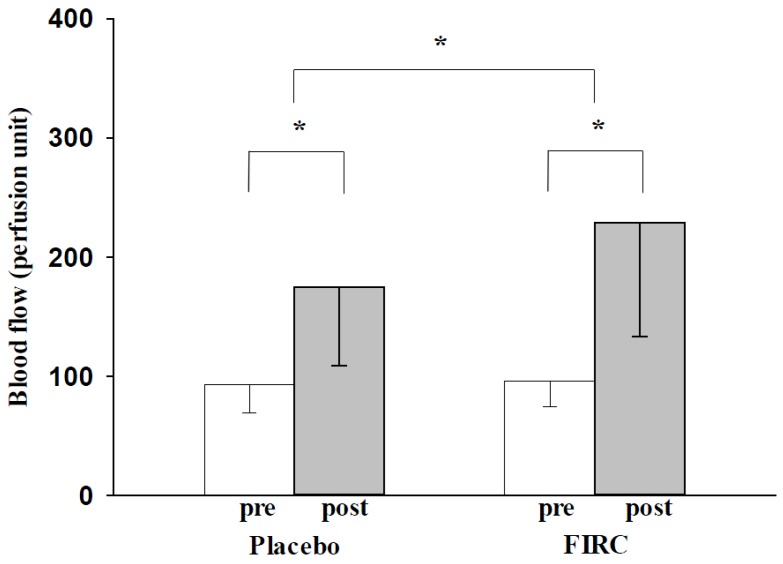
Changes in blood flow of placebo and FIRC groups before and after intervention. Asterisk indicates a significant difference.

Both the FIRC group and placebo group had a significantly increased blood flow of the neck region after the collar test (as shown in Figure 3; FIRC group: pre-96.2 ± 21.3 PU *vs*. post-228.9 ± 95.8 PU, *p* < 0.05; Placebo group: pre-93.5 ± 23.8 PU *vs*. post-174.4 ± 65.7 PU, *p* < 0.05). In the FIRC group, blood flow was promoted significantly better than for the placebo group (*p* < 0.05). Furthermore, the tendency of blood flow alteration was consistent with the temperature changes of the neck, as illustrated. Heat is known to provide thermal energy to dilate blood vessels, improve circulation, and enhance metabolism. Our study found that the FIRC promoted neck metabolism better than a single heating device.

Prior studies have shown that heat provides beneficial effects in pain relief [8,9,10,11]. An increase in blood flow indicated rapid hemodynamic changes and an increasing volume of blood in the neck region. When blood circulation was improved, it could accelerate the excretion of lactic acid, inflammation, and toxin extravasations. These substances are considered to affect muscle cells and neural pathways, and to cause pain [28,34]. Neck disorder caused by muscle fatigue or mood distress may accumulate these pain-producing substances in tissue, and hypersensitivity would be gradually induced by the noxious stimuli from these substances; further pain may result if the pain threshold was lowered by the hypersensitivity [28]. Therefore, an improvement in circulation may relieve painful muscle tension by accelerating the removal of these pain-producing substances. In addition, blood circulation was not merely accelerated hemodynamically from thermal energy. As the hemodynamic effect was promoted, the reaction of the vasodilator neurons was activated, and blood vessels were dilated. The subsequent increase of blood flow volume may evacuate ischemic muscle pain, and rich oxygen and enzymes carried by the bloodstream may remove pain-producing substances as well [1,8,28]. The present study illustrated that FIRC application may improve neck disorders by elevating skin temperature and facilitating blood circulation.

### 2.4. Pain, Anxiety, Depression, and Fatigue

**Figure 4:**
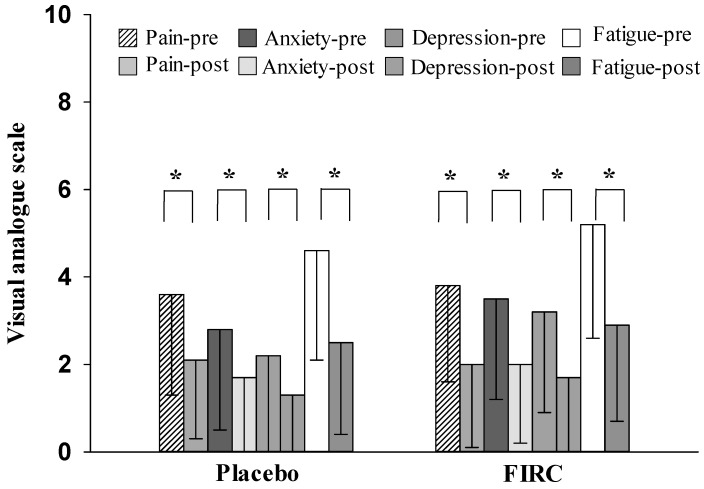
Changes in VAS-Pain, VAS-Anxiety, VAS-Depression, and VAS-Fatigue of placebo and FIRC groups before and after intervention. Asterisk indicates a significant difference.

After applying the collars, both the FIRC and placebo group showed a significant decrease in pain, anxiety, depression, and fatigue (as shown in Figure 4; FIRC group, *p* < 0.05; Placebo group, *p* < 0.05). In addition, no significant differences were present in the changes of these mental parameters between groups (*p* > 0.05). The FIRC and the sham collar both provided their potential thermal effect for pain relief and improvement of mental disorders [35].

The comorbidity of neck pain and mental disorders may be due to neurotransmitters passing through the bloodstream and neural pathway affecting the receptors, the hypothalamic-autonomic-adrenal system, and the hypothalamic- pituitary-adrenal axis [26]. Neck disorders such as cervical neuromuscular syndrome, fibromyalgia in the neck region, and visual display terminal syndrome are common types of neck pain with features of mental disorders and autonomic imbalance involvement [22,26,36]. As noted regarding muscle hardness, neck muscle tension is associated with mental stress, and once the neck muscle rigidity is alleviated, fatigue and psychiatric symptoms are ameliorated [22,31]. The variation of mental status represents the improvement effect of neck disorders, whether it reflects on the pain relief by alleviating muscle tension, or indicates that the pathogenesis of psychologically induced neck pain has been removed. In addition, mood changes may also reflect the degree of satisfaction with the treatment.

Previous studies have demonstrated that a thermal effect relieves pain, ameliorates fatigue, improves depression, and decreases anxiety [8,9,10,11,12,13,14]. In this study, heat energy provided both groups with a significant benefit in mental function. However, the physical evidence of muscle hardness, skin temperature, and blood circulation showed that the application of collars had thermal and non-thermal effects. Specifically, the FIRC showed its superiority in non-thermal effects and had a greater effect on non-thermal biological functions. However, because the capabilities of the placebo were not significant in reducing neck muscle tension, these functions were improved significantly by the FIRC. This may imply that FIRC application was better than the placebo application in the management of neck disorders, although psychologically, the participants’ satisfaction with the application of these two collars showed no significant difference.

This study indicated that the use of the collar appeared to be safe as there were no reported side effects or adverse events. Additionally, the FIRC could significantly improve neck disorders for a short-term application. This short-term investigation could be a foundation for future research with a longer duration. Our study showed no significant difference between the FIRC and the sham collar in moderating the participants’ feelings of pain, fatigue, and mood distress. This may be because our observation period was relatively short. However, our investigation revealed that after short-term use, the heated collar immediately showed its capacity to reduce mental distress. It reflects the reality that when consumers select such products to improve neck disorders, they may be satisfied with the warm/thermal effect after a short period, irrespective of whether the collar contains FIR radiation. 

This study provided a foundation for future research into the development of the collar application as a FIR therapy, and provided valuable information for neck disorder sufferers who may use FIR collars in the future. This study demonstrated that the non-thermal effects of the FIR collars can significantly improve neck disorders. However, the improvement was observed only for a short-term application and the pain relief effect was measured immediately after intervention. The findings of our experiment were in agreement with those of a prior report [37]. A follow-up protocol for long-term FIR application is necessary to evaluate the long-term effects in improving neck pain. Studies providing more physical evidence of neck disorder relief and identification of the biological benefits felt by the participants with different ages are required for future FIR collar development. 

## 3. Experimental Section

### 3.1. Collars of the FIRC Group and Placebo Group

**Figure 5:**
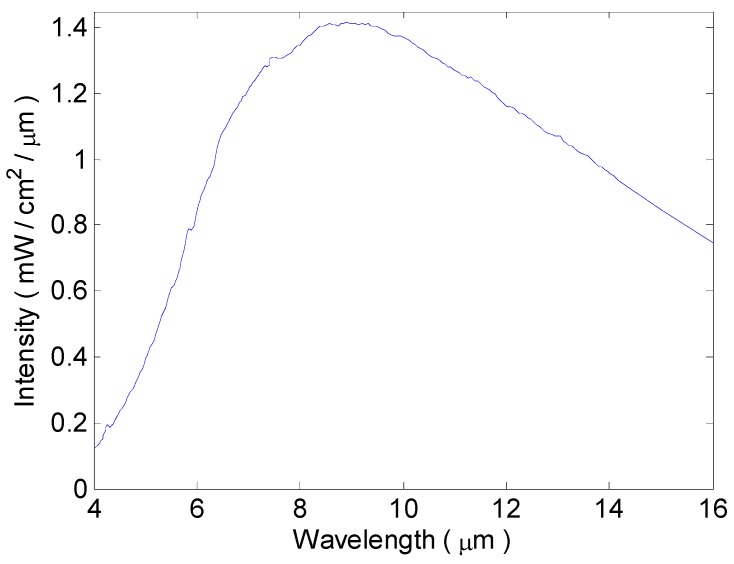
The spectrum of FIR ceramics at 50 °C. The total intensity of the wavelengths between 4 µm and 16 µm is 12.37 mW/cm^2^.

Both the FIRC and placebo group were engaged with collars that had no external difference, but varied in whether the interior fabric contained FIR ceramics. The FIRC group applied the collars of textile fabrics coated with 10 wt % FIR ceramics, whereas the placebo group applied sham collars with no FIR ceramics inside. As in our previous study, the FIR ceramics were powdered with numerous mineral oxides, including aluminum oxide, ferric oxide, magnesium oxide, and calcium carbonate [38,39,40,41,42,43]. The FIR energy was determined using a SR5000 spectroradiometer (CI, Ltd., Migdal HaEmek, Israel) at the Industrial Technology Research Institute, Taiwan. When it was warmed to a temperature of 50 °C, the FIR energy was 12.37 mW/cm^2^ (at a wavelength ranging from 4 to 16 μm) as shown in Figure 5. All collars had a basic heating device (HK55, Beurer GmbH & Co., Ulm, Germany) for elevating their temperature to 50 °C. This was to excite the FIR ceramics in the FIRC group.

### 3.2. Participants 

Sixty volunteers (35 women and 25 men) with neck disorders determined using the Neck Disability Index score of ≥5 were recruited. The mean age of participants was 19.7 years (range 18–28 years). Neck Disability Index is a standardized tool of generic and clinical use; its purpose is to assess the degree of neck pain and disability [21,44]. A score of less than five indicated no disability, and a score of ≥5 indicated that participants experienced not less than mild neck disability [21,45,46]. This criterion suited the target population for FIRC of almost healthy people that show a tendency toward neck pain, because the intention of this study was to assess the FIRC as a convenient and safe home therapy for addressing self-manageable neck disorders. Those with severe neck pain, spine malformation, cervical displacement, or fracture were excluded from the study, as were potential participants with historical cardiovascular and blood diseases, tumors, neurological diseases, and systematic arthritis. These people were referred to a surgeon or other specialists.

### 3.3. Procedures 

This was a randomized, single-blinded, placebo-controlled, parallel-arm trial. Sixty participants were randomly assigned into a FIRC group and a placebo group. Each group had an equal number of participants, with an almost equal distribution of sex between the groups. The experiment was conducted in a conditional laboratory, maintained at a constant room temperature of 20 °C and a relative humidity of 60%. Before the intervention, participants were seated in the laboratory for 30 min. During this time, participants were to modulate their physical and mental status to acclimatize themselves to the room conditions. The participants’ physical measurements and psychometric data were then taken as the baseline. Subsequently, participants wore the FIRC or the sham collars, set at 50 °C, for a further 30 min. All participants were blinded as to which collar they wore. The post-intervention measurements then recorded participants’ physical and mental changes. This study was approved by the Institutional Review Board of Hungkuang University (HK IRB 100-B-004), and the procedures were conducted according to the Declaration of Helsinki. All participants were fully informed and gave written consent.

### 3.4. Physical Monitoring 

Neck muscle hardness, temperature, and blood flow were recorded at baseline and post-intervention. Muscle hardness is a sign of neck pain and an important indicator in the evaluation of neck disorders. Typically, the definition of muscle hardness is the resistance offered by the muscle against perpendicular pressure [47]. A clinically approved muscle hardness meter NEUTONE TDM-N1 (Try-All Corp., Chiba, Japan) was applied to measure neck muscle rigidity [25]. We adopted the middle point between the acromion and the seventh cervical vertebra to measure the hardness of the trapezius muscle. A mean value of 3 repeat measurements was taken to demonstrate the hardness of the neck muscles. The temperature of the neck region was thermographed using a Fluke Ti25 (Fluke Corporation, Everett, WA, USA) [48]. Blood flow of the participants’ neck region was detected using MoorLDI2-IR Laser Doppler Imager (Moor Instruments Ltd., Devon, UK) [49]. Physical recordings were made to evaluate body status and also would be considered in conjunction with variations in mental state.

### 3.5. Participant-Recorded Outcome Measures 

Psychometric measurements were taken to assess participants’ pain intensity, anxiety, depression, and fatigue. These tests were assessed using the standard visual analogue scale (VAS). The VAS for pain measure has good psychometric properties [50,51,52,53,54,55] and is used as the criterion standard against new rating methods [56]. With good reliability and validity for psychometric status [21], VAS is a common clinically-used scale for assessing pain, fatigue, and mood disorders [8,13,57]. Participants were asked to place a mark on a 10-cm horizontal line, which from the left-end indicated no pain and at the right-end indicated extreme pain. Participants reported their mental state at baseline and after the collar intervention.

### 3.6. Statistical Analysis 

The sample size was determined by the analysis of power (G*Power software, version 3.1.9.2, Heinrich-Heine-Universität, Düsseldorf, Germany). A total sample size of 44 can have a medium effect size (*d* = 0.5) with a power of 0.9 at α = 0.05 (two-tailed test) regarding the parameters of outcome measures [58]. Physical and mental measurements taken before and after each participant’s intervention were evaluated using Wilcoxon signed-rank tests. All data were presented as mean ± SD. Furthermore, Mann-Whitney U tests were used to compare the degrees of changes between the groups. A value of *p* < 0.05 was considered significant for all statistical analyses.

## 4. Conclusions

This study demonstrated that the biological benefits of the FIRC were not only from a single thermal effect. Due to the significant change between the groups, the FIRC provided greater therapeutic effects in elevating skin temperature and promoting blood circulation than the single heated collar. Moreover, the FIRC was efficacious in decreasing muscle tension but a single heated collar was not. In addition, there were improvement effects of relieving pain, ameliorating fatigue, improving depression, and decreasing anxiety. FIRC application could be a potential alternative strategy to treat multidimensional neck disorders.
